# Antagonist effects of grain boundaries between the trapping process and the fast diffusion path in nickel bicrystals

**DOI:** 10.1038/s41598-021-94107-6

**Published:** 2021-07-30

**Authors:** J. Li, A. Hallil, A. Metsue, A. Oudriss, J. Bouhattate, X. Feaugas

**Affiliations:** LaSIE UMR CNRS 7356, La Rochelle Université, Av. Michel Crépeau, 17042 La Rochelle Cedex 1, France

**Keywords:** Materials science, Structural materials, Metals and alloys

## Abstract

Hydrogen-grain-boundaries interactions and their role in intergranular fracture are well accepted as one of the key features in understanding hydrogen embrittlement in a large variety of common engineer situations. These interactions implicate some fundamental processes classified as segregation, trapping and diffusion of the solute which can be studied as a function of grain boundary configuration. In the present study, we carried out an extensive analysis of four grain-boundaries based on the complementary of atomistic calculations and experimental data. We demonstrate that elastic deformation has an important contribution on the segregation energy which cannot be simply reduced to a volume change and need to consider the deviatoric part of strain. Additionally, some significant configurations of the segregation energy depend on the long-range elastic distortion and allows to rationalize the elastic contribution in three terms. By investigating the different energy barriers involved to reach all the segregation sites, the antagonist impact of grain boundaries on hydrogen diffusion and trapping process was elucidated. The segregation energy and migration energy are two fundamental parameters in order to classify the grain-boundaries as a trapping location or short circuit for diffusion.

## Introduction

Hydrogen has a strong tendency to segregate or interact with structural defects as point defects, solid solution species, precipitates, dislocations and internal interfaces (inter-phases, grain-boundaries, …). This situation affects the apparent solubility and the mobility of hydrogen and consequently modifies the embrittlement process of metals and their alloys^1^. Many studies^2–6^ support the role of hydrogen state in the control of the properties of hydrogenated materials in a large diversity of cases (hydrogen diffusion, hydrogen induced cracking, electrical properties in semiconductors, catalyze …) and more precisely their mechanical behavior (embrittlement itself). In this context, hydrogen seems to have a larger implication on intergranular fracture^7–13^. While segregation and diffusion of hydrogen at grain boundaries (GBs) have been of great interest for understanding hydrogen embrittlement (HE), the difficulty of hydrogen analysis at grain boundary scale is the limited number of studies carried out in this subject for nickel alloys (see for a review on the subject references^14, 15^). The interactions of hydrogen with grains and grain boundaries are often treated numerically and experimentally so as to separate with a little confrontation^16–18^. Also, at the atomistic scale, there are many works that are rapidly interested in inter-granular decohesion^19–23^ in relation to segregation without a precise focus on the elementary process which occurs at the grain boundary. In contrast, the short circuit of diffusion within the grain boundaries remains a remarkable complex subject that would require more in-depth analysis.

There is little available experimental data of the hydrogen segregation for properly defined GBs. Indeed, adequately characterizing a GB persists to be an engineering challenge, especially the crystallographic orientation and the misorientation angle need to be suitably controlled. For two decades, a large debate was supported by many studies on the possible conflict between the fact that grain-boundaries can be a either a trapping site and/or a short circuit of diffusion^24–28^ in fcc materials. More recently, based on a larger experimental investigation Oudriss et al.^27–29^ have reported that the GBs with low misorientation (Σ1) and a category of “special” grain boundaries (Σ3–Σ29) are usually preferential areas for hydrogen trapping in polycrystalline nickel. In fact, considering their ordered structure, this kind of boundary is accommodated by defects (dislocations, vacancies and more complex organization) that represent potential traps of hydrogen. In opposite, the high angle “random” grain boundaries are considered as the “disordered phase” where the hydrogen diffusion is accelerated in relation to an eased path associated with lower energy barrier. The predominance of one phenomenon over the other depends on the grain boundary energy and the excess of free volume^28^. These results gathered from a correlation between large data of diffusion coefficient and grain boundary character seems to highlight some exceptions^29^ which suggest a considerable diversity of local processes. These mechanisms were improved and discussed an extensive variety of experimental technics with a high spatial resolution which has been reviewed recently^14, 15^. In fcc metals and alloys, the existence of short-circuit diffusion paths of hydrogen was illustrated using the hydrogen microprint technics and Secondary Ion Mass Spectrometry (SIMS) mapping^14, 15, 29–32^. The preferential ingress of hydrogen along grain boundaries was observed by Tanaka et al.^32^ using Ga-FIB-TOF-SIMS to directly visualize deuterium distribution in fcc steel. Microprint technic shows that not all grain boundaries are generally decorated with Ag crystals, which suggest that hydrogen transport capacity of a boundary depends on its microstructural specificity (character, orientation, …)^30, 31^. More recently, Tof-SIMS and EBSD were combined to investigate statistically hydrogen distribution around grain boundaries in polycrystalline nickel^29^. Our results suggest that grain boundaries can be categorized into two families according to how hydrogen is distributed across the grain boundary. The first family designates random grain boundaries which reveal a sharp gap for hydrogen concentration profile across the grain boundaries. The second one is special Σ3^n^ grain boundaries which presents a smooth gradient of hydrogen concentration cross the grain boundary. Despite these new relevant results, it is clear to conclude that actually it stays difficult to demonstrate that hydrogen distribution results in a heterogeneous behavior of diffusion and segregation processes or both. Recent in situ SKPFM analyses using for detecting the local hydrogen distribution around GBs, demonstrate that a random GB with a misorientation of 43° does not significantly facilitate hydrogen diffusion, while a coherent Σ3 twin GB provides a fast path for hydrogen transport^33^. This last result seems in opposite with Oudriss works^28, 29^ and questions the simple view based on random and Coincidence Site Lattice character (CSL—Σ). Additionally, SIMS mapping^29, 34–36^ and recent Atom Probe Tomography observations^37^ highlight a gradient of hydrogen content with a path length higher than the GBs thickness which suggests that hydrogen diffusivity and segregation processes cannot be only discussed in relation to the local structure of grain boundaries.

Based on atomistic simulations, several computational efforts have focused on the hydrogen segregation and diffusion properties and embrittlement consequences for some selected grain boundary in nickel^19, 38–57^. Classically, the grain boundaries are characterized by their energy, excess volume and geometric parameters such as Coincidence Site Lattice; easily accessible data using density functional theory (DFT) or molecular dynamics (MD) simulations^58, 59^. These characteristics are determining factors on the interaction properties of solutes with the GB; however, such global values sometimes appear to be far from representative of local behaviour. According to extensive atomistic simulations, the segregation energy is essential to the understanding of dynamic processes of solute evolution in materials. The minimum segregation energy, commonly used to characterize GB capacity to interact with hydrogen, vary significantly from − 0.04 to − 0.37 eV depending on the GB character^19,45–47, 53^. The effects of last one can be evaluated on the base of literature data for Σ3(111) (− 0.04 eV)^45, 46^, Σ3(110) (− 0.21 eV)^46^, Σ3(221) (− 0.21 eV)^46^, Σ3(112) (− 0.24 eV)^46^, Σ5(012) (− 0.23 to − 0.37 eV)^19, 45, 48–51^, Σ5(001) (− 0.16 eV)^52^, Σ5(310) (− 0.32 eV)^54^, Σ9(221) (− 0.2 eV)^46^, Σ99(557) (− 0.15 eV)^46^and Σ17(140) (− 0.34 eV)^54^. These values can be significantly modified as a function of the conditions applied to atomistic calculations (size of the box, DFT or EAM potential…) but globally the minimum segregation energy increases with the increases of the CSL index. Hallil et al*.* suggest that for Σ3 GBs, that the GB character (energy, and excess volume) can be treated by the notion of the inclination angle ϕ between the two symmetrical tilt grain boundaries (STGB): coherent twin boundary (CTB) and symmetrical incoherent twin boundary (SITB) configurations^46, 60^. Energy and excess volume expands with ϕ and at the same time the minimum segregation energy of hydrogen grows^46^. Based on MD/MC simulations, larger systems can be investigated. On the other hand, Moody et al.^47^ have pointed out that the hydrogen concentration is enhanced in tilt Σ9(221) high energy grain boundary in nickel. More recently, Brien and Foilles^43^ have studied the hydrogen segregation in inclined Σ3 〈110〉 nickel GBs using the hybrid MC/MD and an analytic segregation model. The maximum concentration of hydrogen occurs at the boundary at the inclination with the highest enthalpy. This result also gives a correlation between the hydrogen segregation and the GBs energy since the GB energy amplifies with the inclination angle for the nickel GB Σ3 〈110〉. The hydrogen segregation phenomenon is more pronounced for high energy GBs which may be explained by the high excess volume for these GBs. All these outcomes suggest a correlation with the geometric and energetic configuration of grain boundaries and segregation properties, but the physical bases of this relationship stay ambiguous. More recently, the local state was considered in some nickel grain-boundaries^46, 56^. Some correlation seems to be possible between the local deformation of hydrogen segregation volume defined by polyhedrons using the Voronoi tessellation method. These studies suggest that elastic dilatation and distortion deformation of the site is partially responsible for the segregation energy. Based on these considerations, some authors have used a continuum approach to evaluate the impact of the elastic field associated with GBs on segregation processes^61^. The respective contribution of short and long-range stress continues to be an open question.

The roles of grain boundaries (GBs) in hydrogen diffusion processes were determined from density functional theory calculations by some authors in fcc metals^51, 62, 63^. The energy barriers along the diffusion path towards and within GBs has been related for Σ3 〈110〉 {111} and Σ11 〈110〉 {113) in fcc Fe-γ^62^, and for Σ3 〈110〉 {111}^51, 63^, Σ5 〈100〉 {210}^51, 63^, Σ5 〈100〉 {310}^63^, Σ11 〈110〉 {113}^63^, Σ25 〈100〉 {430}^63^ and Σ41 〈100〉 {540}^63^ in fcc Nickel. In fcc Fe-γ, the Σ3 GB repels hydrogen and the Σ11 offers an easy diffusion path parallel to the GB plane^62^. In fcc nickel, the Σ3 and Σ11 present a quite similar diffusion behavior tho the bulk^63^ and Σ5 GBs exhibit low-barrier paths to facilitate hydrogen diffusion along the GBs^51, 63^ while Σ25 and Σ41 exhibit high-barrier regions which suggest a slower diffusion of hydrogen than the bulk^63^. The authors suggest that a trapping model in relation with the dislocation density is sufficient to relate these data^63^. Despite these appreciable results, a minor confrontation was proposed in the literature between hydrogen diffusivity and segregation capability of GBs which doesn’t offer the opportunity to clarify the trapping process inside the GBs.

Despite numerous experimental and numerical studies, short-circuits of diffusion and trapping processes within grain boundaries in fcc metals and alloys remain a complex subject that is still poorly understood. Furthermore, the confrontation of experience and numerical works has not been currently used in this subject which reduces the quality of the interpretations.

In the present work, a substantial effort was made to gain further understanding of the key issues of hydrogen segregation and diffusion processes near GBs. The hydrogen/grain-boundaries interactions have been examined for four different configurations of nickel bi-crystal systems to question a considerable variety of grain boundaries energy and excess volume. The hydrogen mobility and trapping process have been investigated based on the electrochemical hydrogen charging technique and on atomistic simulations using an Embedded Atom Method (EAM) potential. The confrontation of both technics allows to elucidate some relevant queries on the contribution of grain-boundary geometry to the mobility and trapping of hydrogen. The segregation process is discussed in relation to the systematic determination of short and long-range elastic distortions and the short-circuit of diffusion process is clarified with a confrontation of the different diffusion paths and the segregation energy of each grain boundaries considered. Both aspects offer new insight to disclose the impact of grain boundaries on some physical properties.

## Results

### Some remarkable results from experimental works

**Figure 1:**
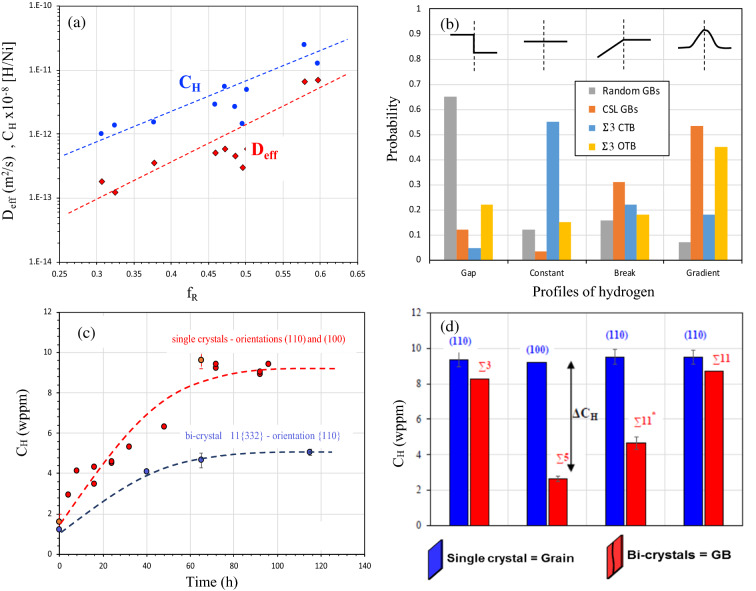
Experimental data collected on the diffusion and solubility in nickel single and polycrystalline pure metal. (**a**) Effective diffusion coefficient D_eff_ and hydrogen concentration C_H_ as a function with the fraction of random grain-boundaries f_R_ (data collected with Oudriss et al. works^28^ electrochemical permeation for the same conditions and for different grain sizes between 10 nm to 200 μm). (**b**) Probability to obtain different gradient profiles of hydrogen concentration near GBs obtained by ToF–SIMS (data^29^ revisited, CTB Coherent Twin Boundary Σ3, Other Twin Boundary Σ3). (**c**) Hydrogen concentration C_H_ *versus* charging time for single crystal and Σ11-{332} bi-crystal with the hydrogen ingress orientation along 〈 110 〉 (hydrogen charging at the cathodic current density of 5 mA/cm^2^ in 0.1 M NaOH at 300 K). (**d**) Hydrogen solubility C_H_ in single crystals and bi-crystals with different hydrogen ingress orientations: two orientations were studied: {110} and {100} (hydrogen charging at the cathodic current density of 5 mA/cm^2^ in 0.1 M NaOH at 300 K for 3 days, Σ3 (Σ3-{111} CTB), Σ5 (Σ5-{310}), Σ11 (Σ11-{311}) and Σ11 * (Σ11-{332}).

**Figure 2:**
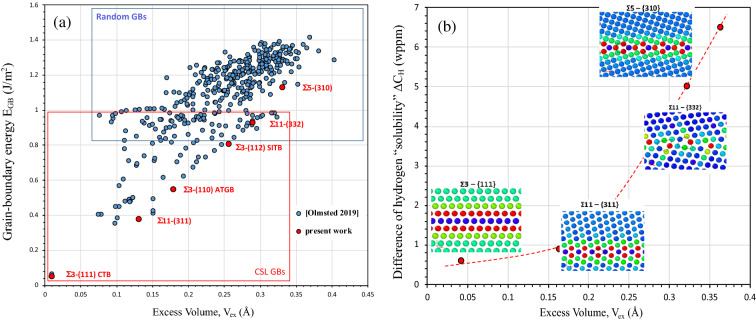
(**a**) Grain-boundaries energy E_GB_ *versus* the excess volume V_ex_ (data from atomistic calculations courtesy to Olmsted et al.^64^). The grains boundaries studies in present work are represented in the picture with red dots. Our calculation has been performed with the same atomic potential. (**b**) the difference of hydrogen solubility ΔC_H_ between single crystal and bi-crystal with the same hydrogen ingress orientation as a function with the grain boundary excess volume V_ex_ (ΔC_H_ are collected by experimental works, V_ex_ is determined numerically using atomistic code).

Recently, an extensive collection of experimental data was gathered for the polycrystalline nickel to characterize diffusion and trapping processes which occur along grain boundaries^9, 27–29^. This database is first revisited in the present work to introduce some open questions. The effective diffusion coefficient D_eff_ was assessed based on classical Fick’s law to describe hydrogen flux across a polycrystalline membrane obtained during electrochemical permeation test. Using a large range of grain sizes it was possible to modulate the fraction of random grain boundary (*f*_R_) and special grain boundary (*f*_CSL_)^27, 28^. A linear relationship is clearly established between Ln(D_eff_) and *f*_R_ the fraction of random GBs, (Fig. 1a) which illustrates the fact that random GBs can act as a short circuit of diffusion. In opposite, it was suggested that CSL GBs are specific locations for deep trapping based on the fact that the number of trapping sites N_T_ increases as function with the fraction of CSL grain boundaries (*f*_CSL_) (Fig. 11b in Oudriss et al. work^28^). Despite this demonstration of the global impact of both nature of GBs (Special and Random) on diffusion and trapping processes, some questions seem to be open. The separation of Special and Random class of GBs based only on the coincidence lower than Σ29 seems unreasonable considering the energy of grain boundaries (see Fig. 2a as an example). Additionally, the hydrogen concentration C_H_ increases with the fraction of random *f*_R_ which suggests that random GB is also a specific location of trapping. This effect was clearly identified as a consequence of a vacancy cluster formation (SAV) process. A linear relationship with vacancy concentration and hydrogen was identified (C_vac_ = 0.15 × C_H_^28^ which illustrates that the fact that the increase of hydrogen concentration is directly a consequence of vacancy formations without clearly establishing that the formation is directly promoted by random grain boundaries. The antagonist properties of trapping sites and short diffusion paths of random GBs illustrate some ambiguities of the interpretation of experimental data. More recently, we had the opportunity to use both TOF-SIMS and EBSD and combinate their analyses to retrieve the statistical information on the location of hydrogen near the GBs as a function of its character^29^. Initially, these data were only analyzed in term CSL Σ3^n^ and random GBs, but in the present work, we show the opportunity to distinguish the coincident twin boundary CTB (Σ3 {111}) to other CSL boundaries OTG (ATGB, asymmetric tilt grain boundary and SITB symmetric incoherent twin boundary: Σ3 {112}). Figure 1b illustrates the different profiles of hydrogen content observed around GBs after pre-charging and releasing steps which corresponds to a study state. A gap of hydrogen concentration between both adjacent grains is mostly related to random GBs. Consequently, it seems that when hydrogen diffusion occurs along GBs, hydrogen does not cross the GB easily but moves along the GB. In opposite, a constant profile where no significant modification of hydrogen content is mainly related to both grains around GB is observed for coincident twin boundary CTB where no defect and elastic distortion are necessary to accommodate the misorientation between adjacent grains.

For the other CSL Σ3^n^ boundaries OTB, a hydrogen gradient around GBs is observed where a large density of defects is necessary to accommodate the misorientation between two adjacent grains. Additionally, it was reported at a micrometer length scale of the gradient of hydrogen concentration significantly higher than GBs thickness (nanometer)^29^ which suggests the occurrence of the long-range internal stresses near OTB GBs. Considering these statistical results, we note that even though we associate a type of grain boundary type (random or special) with a hydrogen concentration profile, this result is by no means exclusive. This conclusion allows us to develop a work specifically on selected grain boundaries in a large domain of representative GBs. We have followed the hydrogen content as a function of charging time for a specific electrochemical charging condition which corresponds to a thermodynamic system defined by P_H2_ = 800 atm and T = 300 K on two systems single and bicrystal with the same ingress surface. The evolution of hydrogen concentration C_H_ as a function of charging time is presented in Fig. 1c for the {110} and {100} single crystals and bicrystal Σ11{332} with a common hydrogen ingress orientation of {110}. In each case, hydrogen concentration increases with time and reaches a saturation plateau corresponding to an apparent solubility. No significant difference is observed between both single crystals. In opposite the hydrogen content is largely lower in the considered bicrystal (5 wppm instead of 9 wppm for single crystals). We provide the same comparison for three other configurations in Fig. 1d for the stationary state. A very low difference is observed between bicrystal with Σ3 {111} (CTB) or Σ11 {311} in comparison with single crystal with a similar hydrogen ingress orientation. In opposite for bicrystal with Σ5 {310} or Σ11{332} the hydrogen content obtained are lower than the one determined for equivalent single crystals (same hydrogen ingress orientation). To resume, for bicrystals the hydrogen content is lower for bicrystals than single crystal with an intensity defined by ΔC_H_ (difference of hydrogen content between single crystal and bicrystal) which depends on the grain boundary character and suggests that GBs act as a short-circuit of diffusion. The difference ΔC_H_ between bi-crystals and single crystal are questioned in term of the energy of grain boundary and the excess volume V_ex_. Both values are evaluated using atomistic calculation with EAM potential (see method) and confronted with data base of Olmsted et al.^62^ (Fig. 2a). Two domains corresponding to Special or CSL GBs and Random GBs are represented in this figure. Additionally, our studied GBs are defined with red dots. In a specific range of energy and excess volume (intersection between two domains), it is impossible to distinguish both kinds of GBs (CSL or Random). According to the Fig. 2a, the first group of our selected GBs: Σ3 {111} (CTB) and Σ11 {311} corresponds to CSL GBs and the second group: Σ5 {310} and Σ11{332} corresponds to Random GBs. Moreover, for the GBs selected in the present work a linear relation can be found between E_GB_ and V_ex_: ${E}_{GB}\approx 3.32\times {V}_{ex}$ despite the fact that we observed a large scatter in the Fig. 2a. The possible correlation between the excess volume V_ex_ and the difference of solubility ΔC_H_ is evaluated in Fig. 2b and allows suggesting that a higher disorder in grain-boundary (higher excess volume/Random GBs) promotes the hydrogen diffusion along grain boundaries which offers an explanation of fact that the hydrogen content is largely lower in the second group: Σ5 {310} and Σ11{332} (Random GBs). Based on these experimental data, the understanding of the competition between hydrogen segregation and diffusion along the grain-boundaries stays a challenge, which is questioned using atomistic calculations in present work.

### Segregation energy versus grain-boundary character

**Figure 3:**
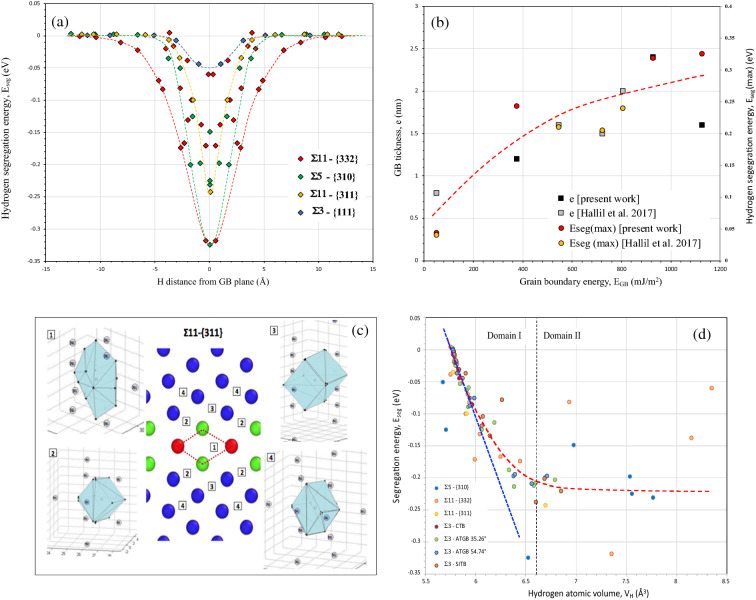
(**a**) Hydrogen segregation energy, E_seg_ as a function of the hydrogen location in the grain boundary core region. (**b**) Grain boundary thickness, e and maximum hydrogen segregation energy, E_seg_(max) *vs* the grain boundary energy E_GB_. (**c**) Segregation position of hydrogen in GB Σ 11-{311} and their volume geometry defined by Voronoi’s method. (**d**) Segregation energy as a function of the hydrogen atomic volume V_H_ for the different locations in the seven grain boundaries studied.

The segregation energy for the different sites in and near the GBs has been determined for the four GBs studied. According to previous works (see as examples^46, 54, 65, 66^), the segregation energy of hydrogen in a specific location in and near GBs is relatively reported to the octahedral site since it is the most stable one in fcc nickel phase (the insertion energy for octahedral site is ${E}_{oct}^{ins}$=0.1775 eV while for tetrahedral site it is equal to 0.586 eV). The energy can be represented as a function of the distance from the GB plane (Fig. 3a). Since a large variety of segregation energy can be obtained for some GBs and specifically for large excess volume (see Σ11-{332} as an example) we characterise this distribution by the maximum of this energy E_seg(max)_ and the thickness, e_GB_ of GBs defined from an energy point of view. e_GB_ characterises a length scale of the impact of GB on the segregation of hydrogen. E_seg(max)_ is obtained in the GBs core and depends on the GB character. Both parameters (E_seg(max)_ and e_GB_) are an increasing function of the grain boundary energy, E_GB_ (Fig. 3b). According to the linear relation between grain boundary energy and the excess volume V_ex_ (Fig. 2a), the last results can be interpreted as a consequence of the disorder impact on the intensity of segregation process and the domain of the occurrence. To question the possible implication of this disorder on the hydrogen segregation site near grain boundaries, we characterise precisely each location of hydrogen segregation firstly by the hydrogen atomic volume, V_H_. This volume defined by neighbouring nickel atoms is calculated using the Voronoi method. The hydrogen atomic Voronoi volume at the bulk octahedral site V_Oct_ is 5.773 Å^3^ and presents a cubic morphology. All segregation sites contiguous to the GB region have a higher atomic volume and a more stable segregation state (Fig. 3d). Concerning the lower energy GBs, the segregation energy E_seg_ for the majority of sites is a quite linear function of the hydrogen atomic volume, V_H_ (domain I, blue curve). The lower energy GBs presents a lower excess volume (Fig. 2a) which corresponds to a large number of sites with potential low hydrogen atomic volume. Consequently, the linear part of the evolution of segregation energy *versus* hydrogen atomic volume (Fig. 3d) is mainly representative to segregation sites of lower energy GBs. However, this linear relationship is not available for high energy GBs with an atomic volume above 6.6 Å^3^ (domain II) in accordance with our previous work related to Σ3^n^ special GBs^46^.

**Figure 4:**
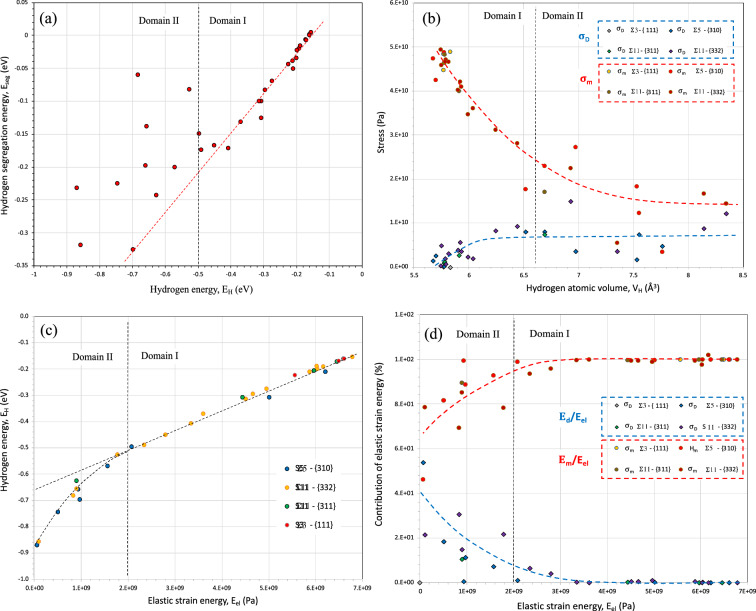
(**a**) Hydrogen segregation energy E_seg_ *versus* hydrogen energy E_H_. (**b**) Hydrostatic σ_m_ and deviatoric stress σ_D_ as a function of hydrogen atomic volume V_H_. (**c**) Hydrogen energy E_H_ *vs* the elastic strain energy E_el_ (per unit of volume) (**d**) Contributions to the hydrostatic, E_H_ and deviatoric, E_D_ parts of energy to the elastic strain energy E_el_.

In domain II, a quasi-plateau is reached in terms of energy (− 0.22 eV) with a large scatter. Consequently, the hydrogen atomic volume seems to be insufficient to question the elastic energy contribution to the segregation energy which allows in the following to consider the morphology of the different sites. The segregation sites in the GB region have complicated local geometry structures, thus, we will describe the geometry of all the potential segregation sites in GBs core in detail. An illustration of this approach is shown in Fig. 3c where the segregation positions and their volume geometry are presented for the Σ11-{332} GB. The position numbers are ranked from the most to less stable segregation energy. According to the Voronoi tessellation, the hydrogen volume at the octahedral site in nickel bulk is a cubic form with 14 neighbouring atoms. The closer the hydrogen atoms get to the GB core region, the greater the geometry deformation occurs. Consequently, the segregation energy and the hydrogen volume size are extremely dependent on the local environment. However, a direct relationship cannot be found among these factors. The morphology of the different sites highlights the fact that the deformation of the site is not isotropic for most cases. We will discuss this crucial aspect in the next section. After the consideration of the local volume deformation, the hydrogen atom insertion at different sites can be discussed in terms of local energy of hydrogen E_H_ (the energy of atom i in the EAM method with i = H). This energy is the sum of the kinetic and the potential energy, which differs from the segregation energy E_seg_ only if we have a long-range effect associated with the insertion of hydrogen. Figure 4a shows the relationships between the segregation energy, E_seg_ and the hydrogen atom energy, E_H_. Two domains are clearly observed in relation with the ones defined in Fig. 3d. For low hydrogen atom energy, a linear relation with segregation energy is clearly established (E_seg_ = 0.54 $\times$ E_H_ + 0.0864) which corresponds to a range of low hydrogen atomic volume range V_H_ < 6.6 ${\AA }^{3}$ (domain I). The slope 0.54 of E_seg_ *versus* E_H_ coincides to the ratio between the unstrained atomic volume Ω_Ni_ (10.904 Å^3^) and one of the unstrained octahedral interstitial sites V_Oct_ (5.773 Å^3^). Consequently, the hydrostatic deformation induced by the incorporation of hydrogen is a second order term for the relationship between E_seg_ and E_H_. For a segregation energy equal to zero (E_seg_ = 0 eV), the hydrogen atom energy, E_H_ is equal to 0.16 eV which is quite similar to the value of the insertion energy for octahedral site ${E}_{oct}^{ins}$ (0.1775 eV). For a higher value of E_H_ above − 0.5 eV (domain II) a large scatter demonstrates that probably long-range displacements of the nickel atoms occur in addition to the short-range displacement of the neighbouring atoms. Concerning Fig. 3d, the picture highlights the fact that hydrogen atom is more stable at a deep segregation site with high atomic volume. Actually, we have noticed that a part of the data has a linear evolution (${E}_{seg}\sim A\times \left[{V}_{H}-{V}_{oct}\right]$ with a slope A equal to 0.43). These sites have a similar cubic form with a relatively low hydrogen atomic volume. This linearity disappears when the distortion becomes significant (V_H_ > 6.6 Å^3^) and probably with a long-range impact of this distortion (see the next section). Moreover, instead of domain II, we observed some specific situations as the fact that some sites where hydrogen atomic volume V_H_ is lower than the one of the octahedral interstitial sites V_Oct_ (5.773 Å^3^). These situations have been recently related near a free surface of nickel single crystal^67^.

### Segregation energy versus elastic energy

As previously highlighted in different configurations, the insertion of a solute in a specific location induces a geometric transformation which cannot be only described as a volume change (see as examples^16, 46, 68–73^). The modification of the geometric configuration of the volume defined by Voronoi’s method results in an equilibrium with the different interactions between the neighboring atoms of the solute. The contribution of the elastic strain energy of this process can be evaluated by considering the respective effect of hydrostatic strain (change of the volume) and shear strain (change of the morphology, deviatoric part of strain). In order to investigate the contribution of the elastic strain energy to the segregation process the elastic dipole tensor P_ij_ is used to describe the equilibrium in a continuum solid of a point-force distribution^46, 73–75^. The elastic dipole tensor characterizes the changes for both the volume and the shape of the interstitial site during the relaxation procedure after the incorporation of the solute in the lattice. From lattice point defect distortion theory, the dipole tensor is given by:

1 $${P}_{ij}=\sum_{m}{f}_{j}^{m/sol}{r}_{i}^{m}$$

where ${f}_{j}^{m/sol}$ is the force exerted from the solute surrounding atoms m and ${r}_{i}^{m}$ is the atomic displacement of atoms. In a first approach, the lattice distortion around the solute is supposed to occur mainly on the insertion site closest to the neighboring atoms. Thus, in isotropic medium, the stress $\sigma_{ij}$ and the elastic strain energy E_el_ by unit volume induced from the insertion of the hydrogen interstitial are related to the dipole tensor^46^:

2 $${{\sigma }_{ij}=\frac{{P}_{ij}}{{\mathrm{V}}_{H}}={\sigma }_{m}{\delta }_{ij}{+\sigma }_{ij}^{d} and E}_{el}={E}_{el}^{m}+{E}_{el}^{d}=\frac{1}{2B}{\left({\sigma }_{m}\right)}^{2}+\frac{1}{6G}{\left({\sigma }_{d}\right)}^{2}$$

where ${\sigma }_{m}$ and ${\sigma }_{d}$ are respectively the hydrostatic stress ($\frac{1}{3}\left[{\sigma }_{11}+{\sigma }_{22 }{+ \sigma }_{33}\right])$ and the deviatoric stress defined as Von Mises equivalent stress ($2{\sigma }_{d}^{2}={\left({\sigma }_{11}-{\sigma }_{22}\right)}^{2}+{\left({\sigma }_{11}-{\sigma }_{33}\right)}^{2}+{\left({\sigma }_{33}-{\sigma }_{22}\right)}^{2}+6({\sigma }_{12}^{2}+{\sigma }_{23}^{2}+{\sigma }_{13}^{2}))$. B and G are the bulk and shear modulus respectively. ${E}_{el}^{m}$ and ${E}_{el}^{d}$ represent the dilatational and distortion energies. The evolution of hydrostatic and deviatoric part of stress field as a function of hydrogen atomic volume is illustrated in Fig. 4b. Two behaviors can be distinguished: in domain I, for a lower value of V_H_ below 6.6 Å^3^, the hydrostatic component is predominant, whereas in domain II both stresses have the same order of intensity. The direct consequence of this result is observed in the correlation between the local energy of hydrogen E_H_ and the elastic strain energy E_el_ (Fig. 4c). This one illustrates a change of behavior between both domains defined previously. In domain I, a linear regime between both energies is observed with a transition around a strain energy density of about 2 GPa. Below this value a non-linear evolution is established and referred to as domain II. As it is illustrated by the Fig. 4d, the elastic strain energy E_el_ is dominated by the hydrostatic stress for the first linear regime (${E}_{el}\approx \frac{1}{2B}{\left({\sigma }_{m}\right)}^{2}$ in domain I, E_el_ > 2 GPa, E_H_ >  − 0.5 eV and V_H_ < 6.6 Å^3^) while the other regime (domain II, E_el_ < 2 GPa, E_H_ <  − 0.5 eV and V_H_ > 6.6 Å^3^) is due to the contribution of the deviatoric and hydrostatic stresses with a similar contribution for the large volumes.

Assuming that local energy of hydrogen is dominated by the elastic strain energy induced by the insertion of the solute, the slope of linear behavior observed in domain I between hydrogen energy, E_H_ and the elastic strain energy per unit volume, E_el_ (Fig. 4c) is directly associated with an apparent hydrogen volume ${V}_{H}^{ap}$ equal to 12 Å^3^. In regime I, the hydrogen volume V_H_ stays equal to the hydrogen volume of the bulk octahedral interstitial site (5.773 Å^3^) with only a difference lower than 10%. The discrepancy observed between ${V}_{H}^{ap}$ and V_H_ is directly associated with the fact that firstly (Eq. 2) we do not consider the impact of hydrogen on the elastic properties. The degradation of elastic properties correlated to the incorporation of hydrogen is known well documented in the literature^17, 73, 76–81^. Based on the linear-elastic theory of the equilibrium between interstitial solute and surrounding metal lattice, the apparent bulk modulus B* can be determined considering the Eshelby’s inclusion model in case of isotropic elastic properties of a solid^76, 77^: ${B}^{*}=B\left[\frac{2(1-2\upsilon )}{(1+\upsilon )}\right]$. Consequently, considering elastic strain energy E_el_ dominated by the hydrostatic stress with the bulk modulus correction we find ${V}_{H}^{ap}=6.5$ Å^3^ which is in accordance with V_H_ obtained in regime I (between 5.7 to 6.4 Å^3^). In agreement with the Fig. 4c, this result indicates that the local energy of hydrogen E_H_ verifies that:

3 $${E}_{H}={E}_{el}^{m}+{E}^{chem}$$

with a dilatational energy equal to: ${E}_{el}^{m}=\frac{(1+\upsilon )}{4B(1-2\nu )}{\left({\sigma }_{m}\right)}^{2}{V}_{H}$.

The second term of the equation Eq. (3), ${E}^{chem}$ is the electronic contribution or “chemical” term which corresponds to the embedding energy due to the distortion of chemical bonds near the solute (${E}^{chem}$= − 0.653 eV). Equation (3) can be extended in regime II, considering the increase of the hydrogen atomic volume and the implication of distortion part of the elastic energy ${E}_{el}^{d}$. For the last component, we also consider the degradation of elastic properties with hydrogen (${G}^{*}={3B}^{*}\left[\frac{(1-2\upsilon )}{2(1+\upsilon )}\right]$). In domain II, V_H_ increases with the decrease of the elastic energy to reach a value near 8.2 Å^3^. This value is very close to the atomic volume of hydrogen localized in a vacancy ${V}_{vac}^{H}$ ($\sim$ 8.66Å^3^)^46^ where the contribution of elastic energy is negligible instead of an electronic contribution in accordance with the present results^82^. Equation (3) can be rewritten considering the previous comment as:

4 $${E}_{H}={E}_{el}^{m}+ {E}_{el}^{d}+{E}_{chem}$$

with $${E}_{el}^{d}=\frac{1}{6{G}^{*}}{\left({\sigma }_{d}\right)}^{2}{V}_{H}$$.

This equation predicts that for total elastic energy ${E}_{el}{=E}_{el}^{m}+ {E}_{el}^{d}=0 eV$ that ${E}_{H}={E}_{chem}$ = − 0.653 eV which is higher than the one related in Fig. 4c in domain II (− 0.85 eV). This difference suggests that the contribution of the electronic component of energy increases in domain II when the hydrogen volume increases to a value near ${V}_{vac}^{H}$. In the present work, the elastic contribution of the energy of hydrogen E_H_ is between 0.286 eV and 0.082 eV in regime I and lower than 0.082 eV in regime II. The main effect is consequently observed in regime I where the hydrostatic strain contribution is predominant and has the same order of magnitude as the chemical contribution.

**Figure 5:**
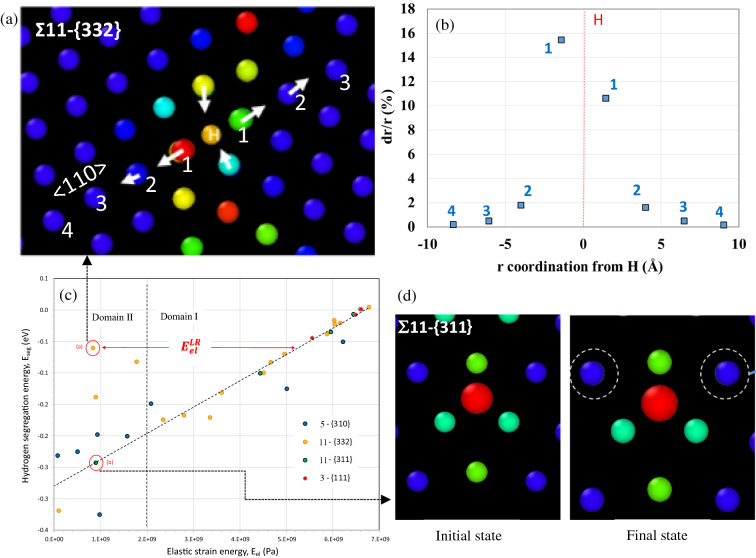
The loss of the linear relation between the segregation energy, E_seg_ and the elastic strain energy E_el_(c) is directly a consequence of the anisotropy of the displacement field and “long range” elastic distortion. (**a**) An illustration of the anisotropy of the displacement field along 〈110〉 near a Σ11-{332}. (**b**) The relative radial displacement dr/r along 〈110〉 as a function of the r coordination from H (corresponding to the Σ11-{332} illustrated in (**a**)). (**d**) Moderate quasi-isotropic short range displacement for a position of hydrogen which respects the linear relation of E_seg_ *versus* E_el_ (see picture (**c**), GB Σ11-{311}).

Both Figs. 3d (${E}_{seg}$ *vs* ${V}_{H}$) and 4a (${E}_{seg}$ *vs* ${E}_{H}$) clearly demonstrate that a significant number of insertion sites do not verify the relationships previously defined. These situations correspond to sites of the grain-boundaries with higher energy and excess volume (Σ11-{332} and Σ5-{310}). The fact that we cannot find a relation with ${E}_{seg}$ and ${E}_{H}$ or ${V}_{H}$, suggesting that a significant part of the segregation energy depends on the long-range elastic distortion. To support this idea, two situations are illustrated in Fig. 5. The first one corresponds to the location (a) where a long-range displacement is observed along the axis 〈110〉 and a short-range displacement in any other directions (Fig. 5a). The profile of the radial deformation is shown in Fig. 5b with a length scale of around $\sim$ 8 Å and an amplitude of 16%. The first neighbor’s energy (1 to 4) corresponds to a long-range elastic interaction ${\mathrm{E}}_{\mathrm{el}}^{\mathrm{LR}}\sim 4.3 {10}^{9}\mathrm{ Pa}$ (or 0.16 eV) which needs to be integrated in the total strain energy density. With this contribution the location (a) follows the linear curve ${\mathrm{E}}_{\mathrm{seg}}$ *vs* ${\mathrm{E}}_{\mathrm{el}}$ defined in domain I with ${E}_{el}={E}_{el}^{d}+{E}_{el}^{m}+{\mathrm{E}}_{\mathrm{el}}^{\mathrm{LR}}$. The second situation illustrated in Fig. 5d is the location (b) in domain II which verifies the linear curve ${\mathrm{E}}_{\mathrm{seg}}$ *vs* ${\mathrm{E}}_{\mathrm{el}}$ without any addition of long-range elastic interactions term because quasi-isotropic short-range displacements are only observed. Consequently, the large scattering observed in Fig. 4a results from the long-range elastic energy between 0.08 eV and 0.44 eV.

### Diffusion paths

**Figure 6:**
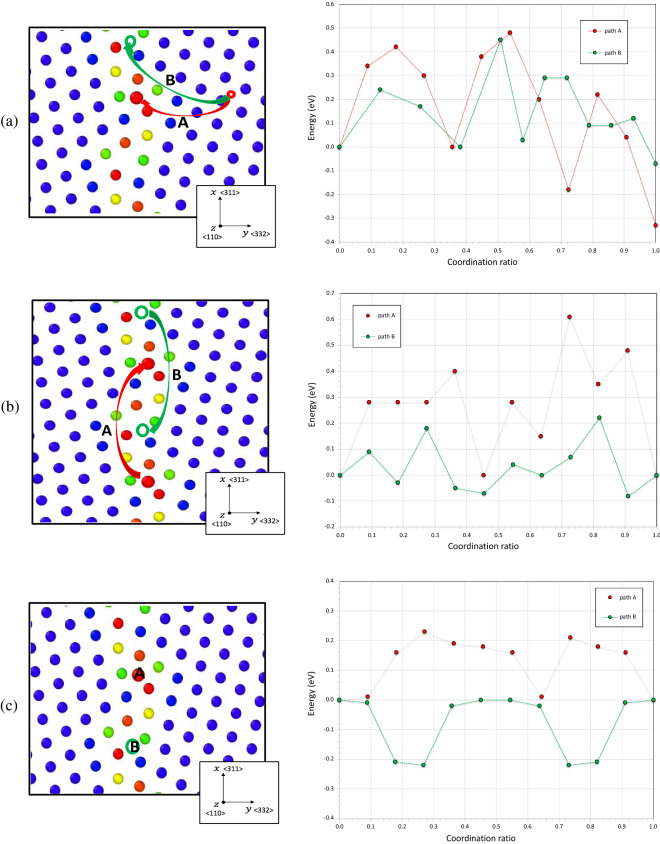
Energy barriers and minimum energy path between the known initial and final states for the GB Σ11–{332}. (**a**) Diffusion paths perpendicular to the grain boundary y =  〈332〉. (**b**, **c**) diffusion paths along the grain boundary respectively for x =  〈311〉 and z =  〈110〉. A is the path between the most stable segregation positions and the bulk. B is the path between the highest volume positions and the bulk.

The short circuit of diffusion along GBs can be discussed as a function with a reference state defined as a diffusion path of the perfect crystal. The nudged elastic band method (NEB) method has been used to calculate the Minimum Energy Paths (MEP) and their associated energy barriers (Table 1). Several stable segregation sites in GBs were looked upon for three principal path directions: (i) the hydrogen atom moves from the bulk site to the GB site (Fig. 6a), (ii) the hydrogen atom moves between two GB sites along the GB plane (Fig. 6b), (iii) the hydrogen atom moves between two GB sites along the tilt axis (Fig. 6c). The first investigation of the migration energy ${E}_{m}$ has been carried out in the perfect nickel crystal. For the easier path, the hydrogen atom moves between octahedral sites (O) through a metastable tetrahedral site (T) in nickel bulk and the energy barriers, ${E}_{m}^{0-T}$ is equal to 0.47 eV in accordance with the DFT works: $\sim$ 0.46 eV^83^ and $\sim$ 0.41 eV^84^. This migration energy is defined as the reference state, the hydrogen atom is able to move faster if the energy barrier is lower than this reference energy. The movement of a hydrogen atom in the GB core and cross the GB is illustrated in Fig. 6 where it is given the MEP and the energy barriers of NEB calculations. The energy barrier is calculated with the initial position energy as the reference point. We have considered several positions such as the most stable segregation sites (type A) and the highest volume sites (type B). A and B positions are the same only for the GBs Σ11-{311} and Σ3-{111} (CTB). In each situation, we have considered the forward (${E}_{m}^{F}$) and backward (${E}_{m}^{B}$) paths. All the energies are reported in Table 1.

**Table 1:** Energy barriers for the most stable segregation position A and the highest volume position B along the different directions X; Y and Z. The forward (${E}_{m}^{F}$) and backward (${E}_{m}^{B}$) paths are differentiated.

Grain-boundaries	Segregation site	X, along GB	Y, across GB	Z, along GB
Σ3-{111} CTB	A/B	${\text{E}}_{{\text{m}}}^{{\text{F}}}$ = ${\text{E}}_{{\text{m}}}^{{\text{B}}}$ = 0.52 eV	${\text{E}}_{{\text{m}}}^{{\text{F}}}$ = ${\text{E}}_{{\text{m}}}^{{\text{B}}}$ = 0.46 eV	–
Σ3-{112} SITB	A/B	${\text{E}}_{{\text{m}}}^{{\text{F}}}$ = ${\text{E}}_{{\text{m}}}^{{\text{B}}}$ = 0.6 eV	${\text{E}}_{{\text{m}}}^{{\text{F}}}$ = 0.42 eV/${\text{E}}_{{\text{m}}}^{{\text{B}}}$ = 0.65 eV	–
Σ11-{311}	A/B	${\text{E}}_{{\text{m}}}^{{\text{F}}}$ = ${\text{E}}_{{\text{m}}}^{{\text{B}}}$ = 0.51 eV	${\text{E}}_{{\text{m}}}^{{\text{F}}}$ = 0.47 eV/${\text{E}}_{{\text{m}}}^{{\text{B}}}$ = 0.72 eV	${\text{E}}_{{\text{m}}}^{{\text{F}}}$ = ${\text{E}}_{{\text{m}}}^{{\text{B}}}$ = 0.6 eV
Σ11-{332}	A	${\text{E}}_{{\text{m}}}^{{\text{F}}}$ = ${\text{E}}_{{\text{m}}}^{{\text{B}}}$ = 0.61 eV	${\text{E}}_{{\text{m}}}^{{\text{F}}}$ = 0.48 eV/${\text{E}}_{{\text{m}}}^{{\text{B}}}$ = 0.81 eV	${\text{E}}_{{\text{m}}}^{{\text{F}}}$ = ${\text{E}}_{{\text{m}}}^{{\text{B}}}$ = 0.23 eV
	B	${\text{E}}_{{\text{m}}}^{{\text{F}}}$ = ${\text{E}}_{{\text{m}}}^{{\text{B}}}$ = 0.3 eV	${\text{E}}_{{\text{m}}}^{{\text{F}}}$ = 0.46 eV/${\text{E}}_{{\text{m}}}^{{\text{B}}}$ = 0.53 eV	${\text{E}}_{{\text{m}}}^{{\text{F}}}$ = ${\text{E}}_{{\text{m}}}^{{\text{B}}}$ = 0.22 eV
Σ5-{310}	A	${\text{E}}_{{\text{m}}}^{{\text{F}}}$ = ${\text{E}}_{{\text{m}}}^{{\text{B}}}$ = 0.51 eV	${\text{E}}_{{\text{m}}}^{{\text{F}}}$ = 0.46 eV/${\text{E}}_{{\text{m}}}^{{\text{B}}}$ = 0.79 eV	${\text{E}}_{{\text{m}}}^{{\text{F}}}$ = ${\text{E}}_{{\text{m}}}^{{\text{B}}}$ = 0.5 eV
	B	${\text{E}}_{{\text{m}}}^{{\text{F}}}$ = ${\text{E}}_{{\text{m}}}^{{\text{B}}}$ = 0.3 eV	${\text{E}}_{{\text{m}}}^{{\text{F}}}$ = 0.46 eV/${\text{E}}_{{\text{m}}}^{{\text{B}}}$ = 0.7 eV	${\text{E}}_{{\text{m}}}^{{\text{F}}}$ = ${\text{E}}_{{\text{m}}}^{{\text{B}}}$ = 0.45 eV

For the CTB Σ3-{111} according to the migration energy, a hydrogen atom can easily cross the GB (${E}_{m}^{GB,Y}=0.46 eV \sim {E}_{m}^{0-T}=0.47 eV$) but it is slightly slowed down along the GB plane (${E}_{m}^{GB,X}=0.52eV>{E}_{m}^{0-T}=0.47 eV$). In opposite, for the SITB Σ3-{112} according to the migration energy, a hydrogen atom can easily cross the GB (${E}_{m}^{GB,Y}={\mathrm{E}}_{\mathrm{m}}^{\mathrm{F}}=0.42 eV \sim {E}_{m}^{0-T}=0.47 eV$) but the backward path is more difficult (${\mathrm{E}}_{\mathrm{m}}^{\mathrm{GB},\mathrm{Y}}={\mathrm{E}}_{\mathrm{m}}^{\mathrm{B}}=0.65\mathrm{ eV}>{\mathrm{E}}_{\mathrm{m}}^{0-\mathrm{T}}$). Additionally, the migration energy along grain-boundary is slower in the GB core than in the bulk ${\mathrm{E}}_{\mathrm{m}}^{\mathrm{GB},\mathrm{X}}={\mathrm{E}}_{\mathrm{m}}^{\mathrm{F}}={\mathrm{E}}_{\mathrm{m}}^{\mathrm{B}}=0.6\mathrm{ eV}>{\mathrm{E}}_{\mathrm{m}}^{0-\mathrm{T}}$). Consequently, the segregation sites are probably trapping sites in present SITB Σ3-{112}.

For the low energy/excess volume GB Σ11-{311} (CSL GB), the hydrogen atom can move easily from the bulk to the GB core (${\mathrm{E}}_{\mathrm{m}}^{\mathrm{GB},\mathrm{Y}}={\mathrm{E}}_{\mathrm{m}}^{\mathrm{F}}=0.47\mathrm{ eV}={\mathrm{E}}_{\mathrm{m}}^{0-\mathrm{T}}$), but the backward path is more difficult (${\mathrm{E}}_{\mathrm{m}}^{\mathrm{GB},\mathrm{Y}}={\mathrm{E}}_{\mathrm{m}}^{\mathrm{B}}=0.72\mathrm{ eV}>{\mathrm{E}}_{\mathrm{m}}^{0-\mathrm{T}}$) as are the other directions (${\mathrm{E}}_{\mathrm{m}}^{\mathrm{GB},\mathrm{X}}=0.51\mathrm{ eV and }{\mathrm{E}}_{\mathrm{m}}^{\mathrm{GB},\mathrm{Z}}=0.6\mathrm{ eV})$ where the diffusion gets slower in the GB core. This GB has a few numbers of sites for the hydrogen segregation (four sites) but its segregation sites are probably trapping sites.

The Random GB Σ11-{332} is a high energy/excess volume GB (Fig. 6). It has several hydrogen segregations sites in the GB core (14 sites). For the segregation site A, the energy barrier along the X direction is higher than the reference energy in nickel bulk (${E}_{m}^{GB,X}=0.61eV>{E}_{m}^{0-T}=0.47 eV$), in opposite for the segregation site B where we observed a fast diffusion path with lower migration energy (${E}_{m}^{GB,X}=0.3 eV<{E}_{m}^{0-T}=0.47 eV$). A quite similar value for the migration energy along Z direction is obtained for the segregation sites A and B (${\mathrm{E}}_{\mathrm{m}}^{\mathrm{GB},\mathrm{Z}}$ equal 0.23 eV for A site and 0.22 eV for B site) which allows to define this direction as a fast diffusion path. For the cross direction Y, the hydrogen atom can move easily from the bulk to the GB core (${\mathrm{E}}_{\mathrm{m}}^{\mathrm{GB},\mathrm{Y}}={\mathrm{E}}_{\mathrm{m}}^{\mathrm{F}}\mathrm{ is equal to }0.48\mathrm{ eV for A site and }0.46\mathrm{ eV for B site}$), but the backward path is more difficult (${\mathrm{E}}_{\mathrm{m}}^{\mathrm{GB},\mathrm{Y}}={\mathrm{E}}_{\mathrm{m}}^{\mathrm{B}}$ is equal to 0.81 eV and 0.53 eV respectively for A and B sites).

The Random GB Σ5-{310} is the highest energy/excess volume GB in our samples, it has less segregation sites than the GB Σ11-{332} in the GB core (8 sites). Along the X direction, it exists a diffusion path for which hydrogen atom moves faster at the highest volume sites B (${\mathrm{E}}_{\mathrm{m}}^{\mathrm{GB},\mathrm{X}}=0.3\mathrm{ eV}<{\mathrm{E}}_{\mathrm{m}}^{0-\mathrm{T}}=0.47\mathrm{ eV}$). However, the diffusion at the most stable segregation sites A slows down ${(\mathrm{E}}_{\mathrm{m}}^{\mathrm{GB},\mathrm{X}}=0.51\mathrm{ eV}>{\mathrm{E}}_{\mathrm{m}}^{0-\mathrm{T}}=0.47\mathrm{ eV})$ and can be considered as a trapping site. Along the Z direction, the energy barrier higher than the reference energy in nickel bulk and cannot be considered as a new path of diffusion (Table 1). For the cross direction Y, the hydrogen atom can move easily from the bulk to the GB core (${\mathrm{E}}_{\mathrm{m}}^{\mathrm{GB},\mathrm{Y}}={\mathrm{E}}_{\mathrm{m}}^{\mathrm{F}}= 0.46\mathrm{ eV for for A and B sites}$), but the backward path is more difficult (${\mathrm{E}}_{\mathrm{m}}^{\mathrm{GB},\mathrm{Y}}={\mathrm{E}}_{\mathrm{m}}^{\mathrm{B}}$ is equal to 0.79 eV and 0.7 eV respectively for A and B sites) which promotes the stabilization of hydrogen on GB.

## Discussion

The implications of grain-boundary on hydrogen embrittlement are one key understanding to extend the material performance in an aggressive environment. The design of new materials and alloys requires to improve the fundamental interaction of hydrogen with structural features of grain-boundaries. Before any analysis of the intergranular fracture is necessary to understand the mobility of hydrogen in relation to the microstructural features. Commonly as any solutes, the first step is to focus on the segregation process. The general approach of the segregation is based on a differentiation between an insertion site in GB and the one in lattice (octahedral in fcc alloys) in terms of energy and volume^58, 59, 65, 85^. The domain of the insertion is defined as a volume with specific geometry using space tessellation or Voronoi description. Zhou et al*.*^56^ recently argued that the local volume of the site can be analyzed using only five polyhedrons. Our present investigations clearly illustrate the limitation of this description where we observed a large diversity of geometric forms (see complementary data) especially for grain boundaries with the highest excess volume. According to a large set of data, the segregation energy cannot be directly correlated with the variation of the volume (Fig. 3d) without considering the asymmetry of some sites (see Fig. 3c, site 1) and their distortion (deviatoric part of local strain tensor) during the incorporation of the solute. Another aspect needs to be integrated into this analysis in relation to the impact of hydrogen on the atomic configuration around the insertion site, which depends on the distribution of the different sites along the GB. The first aspect can be considered as a short-range interaction while the second one which corresponds to a long-range distortion according to the length scale larger than the size of the site and the in inhomogeneity of the distribution of these sites. According to the thermodynamic approach, Larche et al.^86^ and Kirchheim et al.^87^ highlighted the possible contribution of the hydrostatic and the deviatoric strain energy on the chemical potential. More recently, the contribution of defects and elastic anisotropy have been questioned on the solubility and diffusivity in nickel^16^. The contribution of shear elastic energy far from any defect stays less common feature related in the literature but a few works show clearly the significance of that one^69–71, 73, 88^. The shear or deviatoric elastic strain energy ${E}_{el}^{d}$ can be mostly near the dislocation core and have a large contribution to the trapping energy^70, 71^. In the present work, the respective contribution on elastic strain energy of hydrostatic ${E}_{el}^{m}$ and deviatoric ${E}_{el}^{d}$ strains in grain boundary (Fig. 4b,d) reveals a significant influence of shear strain in domain II for higher hydrogen atomic volume (V_H_ > 6.6 $\AA$^3^). Additionally, no clear correlation can be obtained in this regime between segregation energy and hydrogen atomic volume (Fig. 3d). The second fundamental aspect emerges in a contribution of a long-range elastic distortion ${\mathrm{E}}_{\mathrm{el}}^{\mathrm{LR}}$ to segregation energy as illustrated in Fig. 5. The importance of this contribution was appropriately illustrated for some specific sites (Fig. 5) where the amplitude of this energy offers the opportunity to explain the discrepancy observed between some experimental data and linear relation between the segregation energy E_seg_ and hydrogen energy E_H_ (Fig. 4a).

Consequently, the segregation energy can be formulated as a function of the different contributions (${\mathrm{E}}_{\mathrm{el}}^{\mathrm{d}}$, ${\mathrm{E}}_{\mathrm{el}}^{\mathrm{m}}$, ${\mathrm{E}}_{\mathrm{el}}^{\mathrm{LR}}$, ${\mathrm{E}}_{chem}{,\mathrm{ E}}_{\mathrm{octa}}^{ins}) :$

5 $${\text{E}}_{seg} = \left[ {\frac{{{\text{V}}_{oct} }}{{{\Omega }_{Ni} }}} \right]E_{H} - {\text{E}}_{{{\text{octa}}}}^{ins} + {\text{E}}_{{{\text{el}}}}^{{{\text{LR}}}} \; {\text{with}} \, E_{H} = E_{el}^{m} + E_{el}^{d} + {\text{E}}_{chem}$$

The chemical component of energy, ${\mathrm{E}}_{chem}$ evolves between − 0.653 eV to − 0.85 eV, the short range elastic energy ${E}_{el}^{m}+ {E}_{el}^{d}$ is evaluated between 0.082 eV to 0.286 eV, the long range internal stresses can be reach a value of 0.44 eV and the insertion energy for octahedral site ${E}_{oct}^{ins}$ is equal to 0.1775 eV.

**Figure 7:**
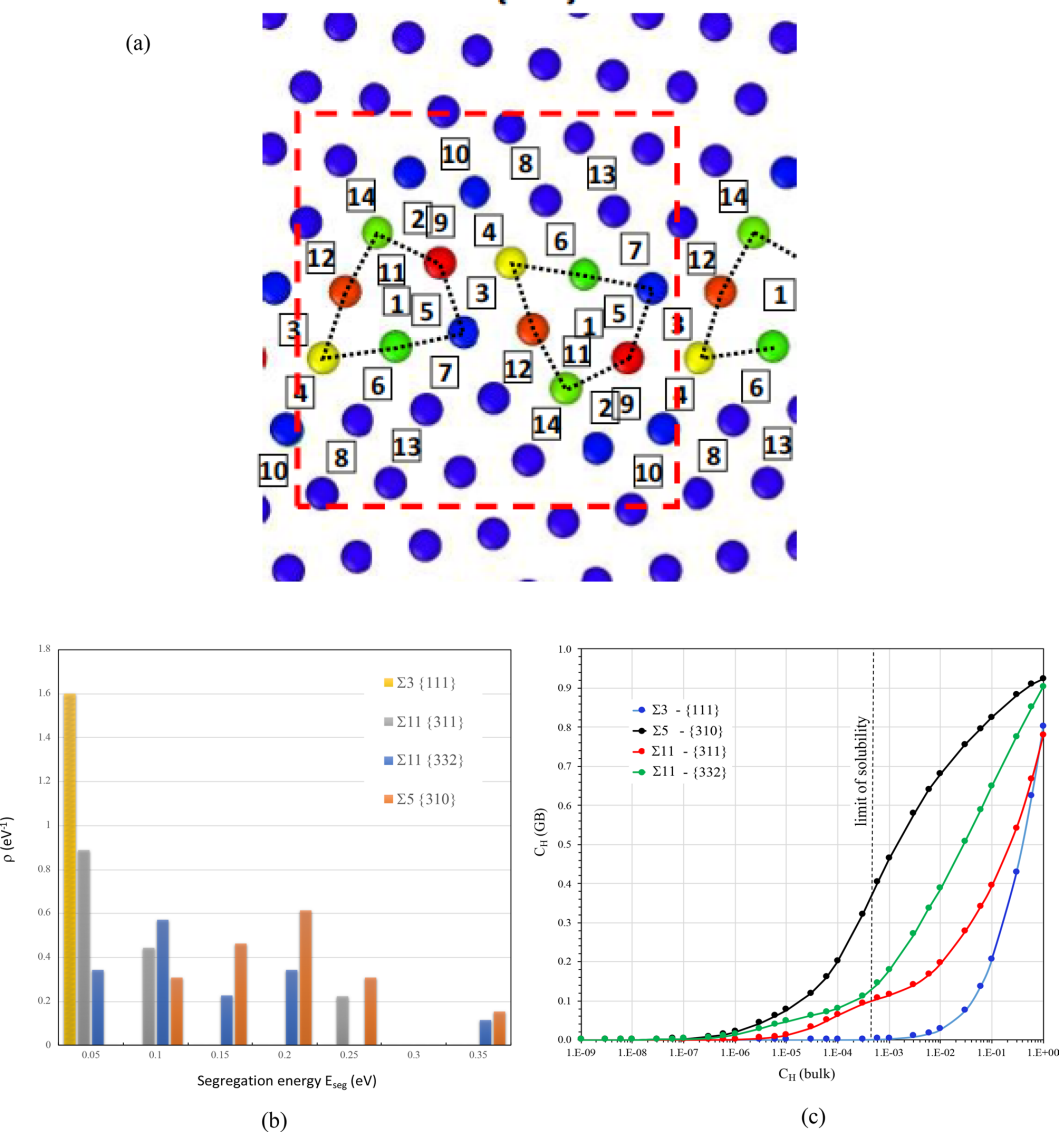
(**a**) The unit cell for GB Σ11-{332} with position details ((31 Ni and 28 sites for hydrogen segregation, 14 sites with different segregation energies). (**b**) The segregation energy density of states (ρ) as a function of absolute value of segregation energy E_seg_ for four GBs. (c) the evolution of hydrogen concentration segregated on GBs is represented as a function with the bulk concentration.

The hydrogen enrichment at the different grain boundaries studied in the present work can be described with a modified analytical Langmuir-McLean segregation isotherm (LML). Instead of initial approach^89^, it was recently answered^90^ the importance to consider the variability of the segregation energy of one grain boundary^91^. Consequently, for each grain boundary, we have determined the number of sites for each segregation energy for one representative motif (see Fig. 7a as an example for Σ11-{332}). Considering the segregation energy density of states, ρ(E_seg,i_) for each GBs, we observed that ρ is maximum for low segregation energy in Σ 3-{111} (Fig. 7b). In opposite, ρ is maximum for the highest segregation energy for Σ5-{310}(Fig. 7b). The segregation energy corresponding to the maximum of ρ follows the relation: E_seg_ Σ3-{111} > E_seg_ Σ11-{311} > E_seg_Σ11-{332} > E_seg_ Σ5-{310}. This order is similar to the one of excess volume of GBs and allows to conclude that Σ5-{310} presents a higher capability to segregation than Σ11-{332} although the number of sites is lower to the one of Σ11-{332}.

According to the Langmuir–McLean segregation isotherm model without solutes interactions^65, 66^, it is possible to establish a relationship between the segregation concentration and the bulk concentration in a binary system. The expression is given in:

6 $${C}_{GB}=\sum_{i=1}^{n}{C}_{max,i}\frac{1}{1+\frac{1-{C}_{bulk}}{{C}_{bulk}}exp\left[\frac{{E}_{seg,i}}{{k}_{B}T}\right]}$$

*Cseg,i* = *CHseg,i/CNi* is the molar fraction of segregation on site i, *Cbulk* = *CH/CNi* is the molar fraction in the nickel bulk. Hydrogen atoms occupy the octahedral site in the nickel bulk, the unit cell of fcc structure gives the maximum saturation *Coct/CNi* = 1. The saturation of segregation *C*max,i of the boundary on a site i depends on the number of the sites with the same segregation energy E_seg,i_. As illustrated in Fig. 7a for Σ11-{332}n = 14 sites can be distinguished. Considering the temperature of 300 K, the evolution of hydrogen concentration segregated on GBs is represented as a function of the bulk concentration (Fig. 7c).

The picture confirms the analysis in terms of the segregation energy density of states. The segregation sites in the high energy GBs such as Σ11-{332} and Σ5-{310} start to be occupied by hydrogen atoms at a very low bulk concentration (*Cbulk* ~ 10^−7^ H/Ni). The GB Σ11-{311} begins the segregation around *Cbulk* = 10^−6^ H/Ni while the GB Σ3-{111} initiates the segregation around *Cbulk* = 10^−3^ H/Ni. The segregation is first initiates at the most stable positions, and then the less steady positions commence to segregate hydrogen atoms when the first position reaches saturation (70%–80% occupation). It has been reported that high local concentrations can act as a seed for local hydrides in nickel^92, 93^ because of the attractive H–H interactions. Moreover, EP/TDS experimental data indicates that the limit of hydrogen solubility in nickel single crystal is around *Cbulk* = 5 × 10^−4^ H/Ni (~ 7.5 wppm at 300 K). Consequently, we compared thus the segregation in different GBs within the range of the bulk concentration between 10^–7^ and 5 × 10^−4^ H/Ni. Hydrogen segregation is stronger in Σ5-{310} than in Σ11-{332}, the segregation concentration is dominated by the most stable segregation position of which E_seg,1_ Σ5-{310} = − 0.3252 eV E_seg,1_ Σ11-{332} = − 0.3188 eV. Additionally, the GB Σ3-{111} acts as the nickel bulk with no significant segregation at all.

Once hydrogen atom is localized in a GB, it is now appropriated to ask now how the atom will move or remain trapped using the evaluation of the migration energies.

The hydrogen atom in all GBs (Σ3-{111}, Σ11-{311}, Σ5-{310} and Σ11-{332}) can easily move from the nickel bulk to the GB core: the forward energy ${\mathrm{E}}_{\mathrm{m}}^{\mathrm{GB},\mathrm{Y}}={\mathrm{E}}_{\mathrm{m}}^{\mathrm{F}}$≈ 0.47 $\pm$ 0.01 eV is quite similar to the migration energy in the bulk ${{\mathrm{E}}_{\mathrm{m}}^{\mathrm{bulk}}=\mathrm{E}}_{\mathrm{m}}^{0-\mathrm{T}}=$ 0.47 eV. GBs such as Σ11-{311}, Σ5-{310} and Σ11-{332} prevent the hydrogen atom from moving from the GB core to the bulk (backward energy ${\mathrm{E}}_{\mathrm{m}}^{\mathrm{GB},\mathrm{Y}}={\mathrm{E}}_{\mathrm{m}}^{\mathrm{B}}$ > 0.47 eV), however Σ3-{111} GB act if the bulk had little influence on this direction (${\mathrm{E}}_{\mathrm{m}}^{\mathrm{GB},\mathrm{Y}}={\mathrm{E}}_{\mathrm{m}}^{\mathrm{B}}$ = 0.46 eV). The energy barrier is able to provide an estimation for the ratio of the diffusion coefficient in comparison to the diffusion in the perfect nickel bulk as it was previously proposed by Di Stefano et al.^51^:

7 $$\frac{{{\text{D}}_{{{\text{GB}}}} }}{{{\text{D}}_{{{\text{bulk}}}} }} = {\text{exp}}\left( { - \frac{{{\text{E}}_{{\text{m}}}^{{{\text{GB}},{\text{i}}}} - {\text{E}}_{{\text{m}}}^{{{\text{bulk}}}} }}{{{\text{k}}_{{\text{B}}} {\text{T}}}}} \right)\; {\text{with}} \, {\text{i}} \in \left\{ {{\text{X}},{\text{Y}},{\text{Z}}} \right\}$$

Two situations can be considered when the backward energy is higher than the one in bulk. The first one corresponds to the segregation of hydrogen to an easy migration path along the grain boundary (directions X and/or Z) where the ${\mathrm{E}}_{\mathrm{m}}^{\mathrm{GB},\mathrm{X or Z}}$ is lower than in the bulk and consequently $\frac{{\mathrm{D}}_{\mathrm{GB}}}{{\mathrm{D}}_{\mathrm{bulk}}}$ > 1. This situation is commonly named a short circuit of diffusion. The second case is a situation where all the migration energies along GB are higher than in the bulk and consequently $\frac{{\mathrm{D}}_{\mathrm{GB}}}{{\mathrm{D}}_{\mathrm{bulk}}}$ < 1. This situation is defined as a trapping configuration.

**Figure 8:**
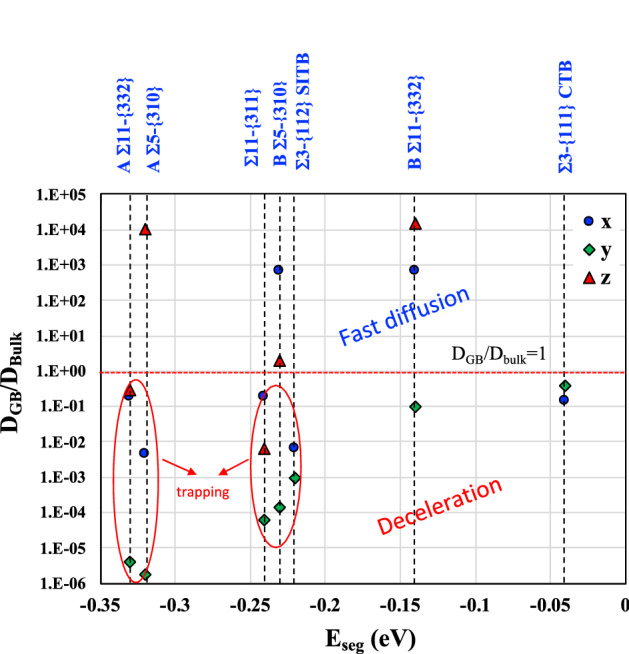
The diffusion coefficient ratio as a function of the hydrogen segregation energy following x, y and z directions for different GBs.

Figure 8 illustrates a distinction (bulk red line) between the accelerated diffusion and the trapping in segregation sites type A (most stable segregation sites) and B (highest volume sites) for the different GBs studied. High energy/excess volume GBs (Σ11-{332} and Σ5-{310}) have one or two accelerated diffusion path(s) where the hydrogen atom moves 10^3^ ~ 10^4^ faster than the diffusion in the nickel bulk. These results are confirmed by our experimental data where for both GBs the hydrogen content in bi-crystals is lower than in single crystal for the same orientation (Fig. 1c,d). Indeed, Σ11-{332} is a GB that have more segregation sites in the GB core with two accelerated diffusion paths, and none of these segregation sites has the trapping behavior (Fig. 8). While GB Σ5-{310} has only one accelerated diffusion path and the deepest segregation site in this GB is a trapping position for the hydrogen atoms (Fig. 8). Σ11-{311} GB do not present any accelerating diffusion paths but only trapping sites in the GB core. Moreover, no significant difference is observed in terms of hydrogen content in single and by-crystals (Fig. 1d). These results allow to conclude that this GB is not a fast diffusion path as CTB Σ3-{111}. Similar results in GBs Σ11-{311} and Σ3-{111} have been reported by Du et al.^62^ in γ-Fe based on their DFT calculations. To conclude, based on a combined analysis of the segregation energy and migration energy of the different potential sites of hydrogen in one GB, it is possible to define one GB as a short circuit of diffusion or trapping location for hydrogen. In perspective, we can enlarge this analysis for a large number of GB configurations considering the similarity in terms of ${\mathrm{E}}_{\mathrm{m}}^{\mathrm{GB},\mathrm{i}}$ and ${E}_{H}$ using atomistic calculation and machine learning^90^.

## Conclusion

We developed a methodology based on the confrontation between experimental hydrogen charging and atomistic modeling to elucidate the competition between hydrogen trapping and short circuit of diffusion along grain boundaries.

Using thermal desorption spectroscopy and electrochemical permeation testing, we measured the diffusivity of hydrogen and trapping energy in single and poly crystals of nickel on a large variety of configurations. Our experimental results are challenged by our atomistic simulations to provide a straightforward understanding of the apparently ambiguous and antagonist effects of grain boundaries between the trapping process and the fast diffusion path. The origin of the acceleration of the diffusivity along grain boundaries is observed when the excess volume increases, which is clarified in terms of migration energies and the distribution of segregation energies. The fast diffusion of hydrogen observed in some grain boundary configurations corresponds to high segregation energy sites and different paths along inter-connected sites of low migration energy. In opposite, the trapping process occurs in grain boundaries of high segregation energy sites and high migration energies. We underline the importance of the elastic energy to global hydrogen incorporation energy in one specific site of grain boundaries. Due to the complex structure of the grain boundary, the distribution of the segregation energy depends not only on the hydrostatic strain energy but also on the deviatoric elastic energy and the long-range elastic distortion which can predominate far from GB. The present work can be extended to a large variety of grain boundaries to allow a more generic relationship between elastic, segregation and migration energies.

## Methods

### Material design and structural characterization

The different nickel samples investigated were high purity single crystals (99.999% purity), bi-crystals (99.95% purity) and poly-crystals/nanocrystals (99.99% purity). Nickel single crystals with three different crystallographic orientations 〈001〉, 〈110〉 and 〈111〉 were provided by Goodfellow. Samples are cylindrical with an 18 mm diameter for nickel 〈001〉 and 11 mm for nickel 〈110〉 and 〈111〉. They were obtained by Bridgman-Stockbarger method with an accuracy of ± 3°. Nickel bi-crystals were made in Laboratory (provided by professor L. Priester (ICMPE) and developed in Mines St-Etienne School). The material for solidification was electrolytic nickel which was remelted under vacuum. The bicrystals were grown from a seed in an argon atmosphere using the horizontal boat method (Chalmers method) of a final length of 14.4 cm at a rate of about 3 mm per hour. The impurity contents are given lower than 0.05 wppm, the sulfur content could be enough to promote intergranular segregation^94^. All samples were then carried out a desulfurization heat treatment at 550 °C for 10 h in flowing hydrogen. Polycrystalline nickel with micrometric grain sizes between 18 and 200 μm was controlled by thermo-mechanical processing at constant annealing temperature for a predefined time in a controlled atmosphere on cold-drawn round nickel rods provided by Goodfellow^95^. The nanometric sizes from 20 to 120 nm were synthesized by electrodeposition using a conventional additive-free sulfamate bath according to the deposition parameters and conditions described previously^96^.

All the microstructures were characterized using Electronic Back Scatter Diffraction (EBSD) and Transmission Electronic Microscopy (TEM). EBSD analyses using an EDAX/ TSL OIM system coupled to an FEI Quanta 200 ESEM- FEG scanning electron microscope were used to characterize the crystallographic orientation (texture, surface orientation) and grain boundaries character (misorientation, coincidence site lattice)^28^. Dislocation densities of each sample were investigated using a JEOL JEM 2011 electron microscope operating at 200 kV. Foils for transmission electron microscopy (TEM) were thinned in a double twin-jet electro-polisher using electrolyte under the conditions described previously^97^. An estimation of the dislocation density of the as-received material gave an average value of less than 10^−10^ m^2^.

### Charging hydrogen and permeation conditions

The electrochemical permeation (EP) test and thermal desorption spectroscopy (TDS) were used to characterize macroscopically the diffusivity and concentration of hydrogen for different conditions. The EP technique introduced by Devanathan and Stachurski^98^ is the main technique used to detect the mechanisms of diffusion and trapping of hydrogen in different microstructures^15, 16, 28^. This technique is composed of two cells separated by a membrane with an exposed surface in contact with an electrolytic solution. The charging side (entry side) of the EP was galvanostatically polarized at a constant cathodic charging current density (5 to 100 mA/cm^2^) in 0.1 M NaOH. The detection side (exit side) of the EP was maintained with a constant anodic potential of ~ 630 mV/SSE in 0.1 M NaOH, and the hydrogen flux (current density) at the detection side was recorded to study the transport of hydrogen through the membrane. The temperature was maintained at 300 K and both solutions were continuously deaerated under argon flux at 1.4 bar. Before the permeation test, both surfaces of the sample were prepared by mechanical polishing up to grade 4000 SiC; the final thickness of the sample was about 200 ± 20 μm.

To quantify the hydrogen concentration and the maximum solubility in nickel, we used Thermal Desorption Spectroscopy (TDS)^15, 28^. These analyses were performed with a Jobin Yvon Horiba EMGA-621W hydrogen analyzer composed of an impulsion furnace system coupled with a thermal conductivity detector. The procedure used consists involved measuring the hydrogen concentration in the pre-charged samples (0.1 M NaOH at 298 K under galvanostatic polarization − 10 mA/cm^2^ with different time) by fusion. For this purpose, after hydrogen charging, the specimen (dimension 8 × 4 × 0.3 mm^3^) were mechanically polished with 5 μm SiC grinding paper, and then cleaned in acetone before introduced into the furnace, where they were instantly heated to 2000 °C and maintained at this temperature for 75 s. The desorbed hydrogen was then detected and analyzed by gaseous Catharometry. The recorded curve corresponds to the amount of hydrogen detected as a function of time. The average concentration of hydrogen in the sample was estimated by measuring the area under the curve.

### Atomistic simulation

**Figure 9:**
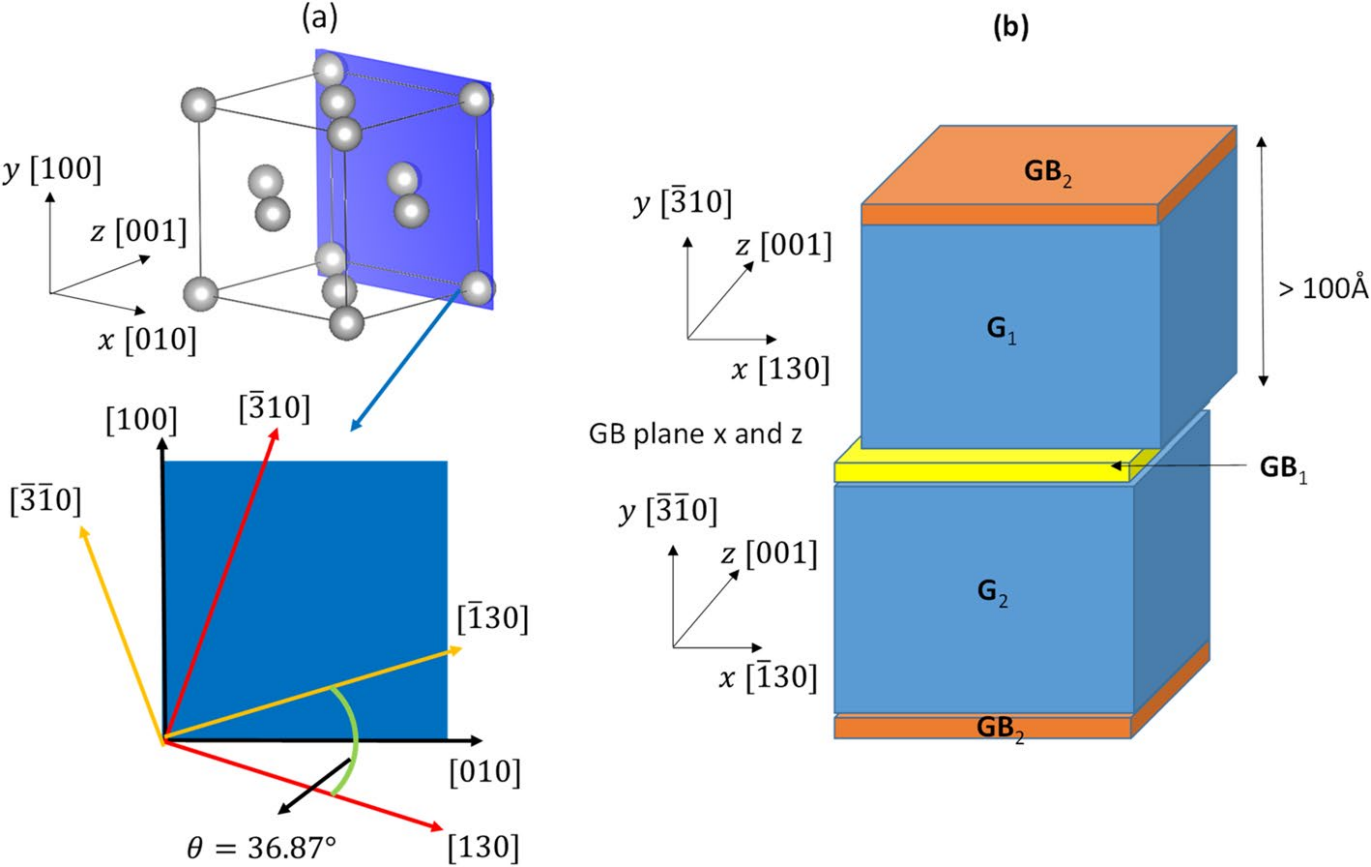
(**a**) GB Σ5-36°87 〈001〉 {310} plane for nickel. (**b**) Schematic of a bi-crystal simulation cell.

The atomistic investigation following the works of Hallil et al.^46, 60^ was carried out using LAMMPS code (Large-scale Atomic/Molecular Massively Parallel Simulator) for MD simulations at 0 K, or more precisely, molecular statics (MS). The atomistic simulations based on the relaxed configuration of atoms are found using the minimization of the total energy with an appropriate interatomic potential at 0 K and 0 Pa. For the H-Ni system, the interatomic potential was established by the embedded-atom method (EAM)^53^. The energy E_i_ of atom i (H or Ni) in the EAM method is given by the following equation:

8 $${E}_{i}={F}_{\alpha }\sum_{i\ne j}{\rho }_{\beta }\left({r}_{ij}\right)+\frac{1}{2}\sum_{i\ne j}{\varphi }_{\beta }({r}_{ij})$$

where F_α_ is the embedding energy function, ρ is the partial electron density contribution, *r*_*ij*_ denotes the distance between atom i and j, φ is the pair potential, α and β are the element types of atom i and j. For a grain boundary in a bi-crystal system, the construction of grain boundary is built by finding the most optimal configuration. An example of a simulation cell for the grain boundary Σ5-36°87 〈100〉 {310} is illustrated in Fig. 9.

θ is the misorientation angle between two identical nickel crystals around the symmetric tilt axis along the grain boundary plane. The direction along the GB plane is designated by the tilt z-axis [001] in the simulation cell and common for both grains. Each GB simulation cell contains two-grain lattices which are characterized by two distinct crystallographic orientations in x and y directions. The GB simulation cells are considered in 3D periodic boundary conditions, this representation provides existence of two GBs in each simulation cell: one in the middle of the simulation cell and on another counting for a mirror image in the bound parts of the simulation cell. The separation distance between each GB is chosen to be large enough so that there will be no energetic interaction between two GB interfaces. A rigid body translation parallel to the GB plane has been applied following x and z-axis, all translational vectors are in a primitive cell of the displacement shift complete (DSC) lattice^59^ and the lattice spacing in the planar directions of each grain is kept constant. The translation of one grain relative to the other yields to a re-arrangement of atoms at the GB plane. After testing hundreds of configurations, the one with the minimum energy at the grain boundary is obtained and the excess volume could be calculated using Voronoi tessellation method implemented in LAMMPS code.

Since the GB structure is well defined, we can insert an atom of hydrogen into the GB. The initial position of the hydrogen atom is at the vicinity of a nickel atom. Then the energy minimization occurs and the hydrogen atom will find its stable position using atomic simulation analysis OVITO software. After determining several stable segregation sites in the core region, we focused on the diffusion paths among these different segregation sites using the nudged elastic band method (NEB)^99^. This method is used to detect saddle points and minimum energy paths (MEP) between the known initial and final states. Transition states of diffusion paths (referred to as images) identify the lowest possible energy while maintaining equal spacing to neighboring images. Once the images have converged sufficiently to the MEP, the image at the highest energy point is allowed to climb uphill along the MEP until it reaches the transition state enabling thus the transition geometry and energy to be accurately defined from the NEB method^98^. We have investigated the MEP and the energy barrier between the most stable segregation positions and the highest Voronoi volume positions (the volume occupied by the hydrogen atom) using LAMMPS code with NEB package. The GB energy is computed as the difference between the total energy of the relaxed GB atoms and the bulk energy in the whole system in GB plane. For a number of atoms *N*_*at*_ in the calculation, the grain boundary energy $E_{GB}$ is given as:

9 $${E}_{GB}=\frac{1}{2}\left[{E}_{GB}^{Ni}\left({N}_{at}\right)-{E}_{Bulk}^{Ni}({N}_{at})\right]/{A}_{0}$$

where ${E}_{GB}^{Ni}\left({N}_{at}\right)$ is the total energy of the relaxed GB, ${E}_{Bulk}^{Ni}\left({N}_{at}\right)$ is the total bulk energy, *A*_*0*_ is the area of the GB plane. $\Delta {V}_{GB}$ the excess volume of a GB can be accessed with (volume variation per unit of GB area):

10 $${\Delta V}_{GB}=\frac{1}{2}\left[{V}_{GB}^{Tot}\left({N}_{at}\right)-{V}_{Bulk}^{Tot}({N}_{at})\right]/{A}_{0}$$

where ${V}_{GB}^{Tot}\left({N}_{at}\right)$ is the volume of GB, ${V}_{Bulk}^{Tot}\left({N}_{at}\right)$ is the volume of bulk.

When the stable configuration of GBs has been established, we started to insert the hydrogen atom in different locations in the GBs. The insertion energy (in some publications called also the adsorption energy) of a hydrogen atom in the nickel lattice ${E}_{H}^{Ins}$ is given in [Eq. (11)]:

11 $${E}_{H}^{Ins}={E}_{Ni+H}^{Tot}-{E}_{Ni}^{Tot}-\frac{1}{2}{E}_{{H}_{2}}$$

${E}_{Ni+H}^{Tot}$ is the total energy of nickel lattice with a hydrogen atom, ${E}_{Ni}^{Tot}$ is the total energy of nickel lattice without a hydrogen atom, ${E}_{{H}_{2}}$ is the chemical potential of the molecular hydrogen and the binding energy of one hydrogen atom can be obtained by the EAM potential in vacuum^99^ ($\frac{1}{2}{E}_{{H}_{2}}$ = − 2.36947 eV). This calculation has indicated that the octahedral site (${E}_{Oct}^{Ins}=$ 0.1775 eV) is more stable than the tetrahedral site (${E}_{Tet}^{Ins}=$ 0.586 eV). Similar results of EAM have been reported by Huang et al*.*^54^. Thus, the segregation energy relative to the octahedral site is written in [Eq. (12)]:

12 $${E}_{H}^{seg}=\left({E}_{GB+H}^{Tot}-{E}_{GB}^{Tot}\right)-\left({E}_{Ni+H}^{oct}-{E}_{Ni}^{oct}\right)$$

${E}_{GB+H}^{Tot}$ is the total energy of GB with a hydrogen atom, ${E}_{GB}^{Tot}$ is the total energy of the GB and ${E}_{Ni+H}^{Oct}$ is the total energy of the nickel lattice with a hydrogen atom at the octahedral site.

### Impact statement

We clearly established the impact of the geometry and the deformation of hydrogen insertion site on the diffusion path and segregation process along grain-boundaries. The analyses offer the opportunity to clearly precise the trapping process on grain-boundary. A well-defined effect of deviatoric and hydrostatic elastic energies on segregation energy was identified at a short-range scale and additionally significant impact of a long-range elastic distortion observed for some GBs configurations.
